# News and views

**DOI:** 10.1111/iwj.14084

**Published:** 2023-01-30

## 1 UNIVERSITY OF NOTTINGHAM

### 1.1 Scientists have discovered a new material that can be applied to diabetic wounds to heal them faster with just one application

Researchers from the University of Nottingham have discovered a new class of polymer that can provide instructions to both immune and non‐immune cells to aid healing in hard‐to‐treat diabetic wounds. The findings have been published in Advanced Materials.

Wound healing is a complex biological process that involves various cell types working together, with a cell type called fibroblasts playing a critical role in forming new tissue required for healing. Diabetes can disrupt these processes in cells making wound healing slow and difficult to treat. This can lead to infection and in extreme cases, the need for amputation.

Experts from the School of Life Sciences and Pharmacy screened 315 different polymer surfaces, examining the different chemical make‐up of each until they identified a polymer type that actively drives fibroblasts and immune cells to promote healing. A team from the School of Engineering made small particles that are decorated with this polymer on their surface. These particles could be directly applied to the wound area.

A polymer is a chemical compound made up of molecules bonded together in long, repeating chains. This structure gives polymers unique properties that can be tailored for different uses. Using polymer microparticles the team showed how this new material, when delivered to a wound on an animal model, produces three times more fibroblast activity over a period of up to 96 h and achieved more than 80% wound closure.

This new polymer could be applied as a coating to standard wound dressings to provide a fast and effective treatment.

Professor Amir Ghaemmaghami from the School of Life Sciences at the University of Nottingham is one of the lead authors on the study and says: “This research is a significant step towards being able to create a new, low cost, effective treatment for diabetic wounds. The results we saw were achieved in just one application, which could be transformative for patients whose current treatment often involves repeated treatments delivered by trained health professionals.”

Professor Morgan Alexander from the School of Pharmacy at the University of Nottingham added: “We have shown the medical potential of novel polymers in previous work; our bacterial biofilm resistant materials are used on urinary catheters in the NHS, showing how this can prevent infection by changing the bacterial cell behaviour at the polymer surface. These polymers also have the potential to be easily applied to dressings, and we are already working with industry partners to develop ways to help wound healing in this way.”

## 2 DUKE UNIVERSITY MEDICAL CENTRE

### 2.1 Scientists find key reason why loss of smell occurs in long COVID‐19

The finding provides an important insight into a vexing problem that has plagued millions who have not fully recovered their sense of smell after COVID‐19.

While focusing on the loss smell, the finding also sheds light on the possible underlying causes of other long COVID‐19 symptoms ‐‐ including generalised fatigue, shortness of breath, and brain fog ‐‐ that might be triggered by similar biological mechanisms.

“One of the first symptoms that has typically been associated with COVID‐19 infection is loss of smell,” said senior author Bradley Goldstein, M.D., Ph.D., associate professor in Duke's Department of Head and Neck Surgery and Communication Sciences and the Department of Neurobiology.

“Fortunately, many people who have an altered sense of smell during the acute phase of viral infection will recover smell within the next one to two weeks, but some do not,” Goldstein said. “We need to better understand why this subset of people will go on to have persistent smell loss for months to years after being infected with SARS‐CoV2.”

In the study, Goldstein and colleagues at Duke, Harvard and the University of California‐San Diego analysed olfactory epithelial samples collected from 24 biopsies, including nine patients suffering from long‐term smell loss following COVID‐19.

This biopsy‐based approach—using sophisticated single‐cell analyses in collaboration with Sandeep Datta, M.D., Ph.D., at Harvard University—showed widespread infiltration of T‐cells engaged in an inflammatory response in the olfactory epithelium, the tissue in the nose where smell nerve cells are located. This unique inflammation process persisted despite the absence of detectable SARS‐CoV‐2 levels.

Additionally, the number of olfactory sensory neurons were diminished, possibly due to damage of the delicate tissue from the ongoing inflammation.

“The findings are striking,” Goldstein said. “It's almost resembling a sort of autoimmune‐like process in the nose.”

Goldstein said learning what sites are damaged and what cell types are involved is a key step toward beginning to design treatments. He said the researchers were encouraged that neurons appeared to maintain some ability to repair even after the long‐term immune onslaught.

“We are hopeful that modulating the abnormal immune response or repair processes within the nose of these patients could help to at least partially restore a sense of smell,” Goldstein said, noting this work is currently underway in his lab.

He said the findings from this study could also inform additional research into other long‐COVID‐19 symptoms that might be undergoing similar inflammatory processes.

## 3 CONVATEC

### 3.1 New survey finds 87% of patients with long‐term health conditions face stigma; nurses believe they lack time and resources to provide adequate support

According to new research, patients with long‐term health conditions face stigma, among a variety of other emotional challenges associated with their health condition. At the same time, survey results indicate that their care teams, particularly nurses, believe unable to provide adequate support due to lack of time and resources. The survey, which was conducted by Wakefield Research and supported by Convatec, included responses from 200 patients or their caregivers and 200 nurses in the U.S.

#### 3.1.1 Challenges facing patients

The survey found that 87% of patients or their caregivers believe some level of stigma associated with their or the person in their care's current health condition. Nearly half (44%) reported believing embarrassed to talk about their current health condition, while 43% felt their health condition is not something that's regularly talked about and is rarely represented in the media.

Almost all (99%) patients and their caregivers say that stigma can negatively impact or slow perceived healing of a patient with a current health condition and 96% of nurses agree that a patient's physical healing can be impacted by stigma.

“We already know that the impact of stigma on our patients cannot be understated,” said Karim Bitar, CEO, Convatec. “These survey results demonstrate why emotional and mental health is a societal health priority today. We need to do more, as an industry, to help prevent stigma among these patients – by showcasing stories and experiences of how our patients live confidently, by providing peer‐to‐peer support, and by making conversations easier between friends, family and care teams.”

While emotional support comes in many forms, a patient's medical team plays an important role, with more than half (56%) of patients reporting that they would like more time with their medical team to believe better supported. Almost all (96%) patients and caregivers would like more information about their health condition—with 53% reporting that they would prefer that information come through conversations with their medical team.

#### 3.1.2 Challenges facing nurses: The care gap

As patients ache for support and in the wake of the pandemic when health care professionals believe more overwhelmed and strained than ever, the survey found that more than 2 in 3 nurses (68%) believe they are unable to fully support their patients and almost all (96%) agree that they need more time, resources and education to fully care for them. According to the survey: 71% of nurses need more time to spend with their patients.

56% believe they need more time to be able to devote to learning and education.

51% believe they are currently lacking the resources they need to share directly with patients to care for them most effectively.

Additionally, while most nurses (82%) believe completely or mostly comfortable speaking with their patients about challenges related to their current health conditions, 60% say they are less than completely comfortable. Of those who are less than completely comfortable, 47% say it's because they lack the quality time to do so.

#### 3.1.3 “Forever caring”: A commitment to close the care gap

Convatec, a global medical solutions and technologies company, today announced a new promise and identity, “forever caring,” which reflects the company's ongoing transformation to become a more care‐centric, agile, and accountable organisation.

“We're listening – and most importantly, we're learning,” Karim Bitar added. “In what has historically been a very product‐focused industry, ‘forever caring' is a commitment to the people we serve – and these very patients, caregivers and nurses who participated in this survey. As we continue to bring to life our vision of pioneering trusted medical solutions to improve the lives we touch, we know that the needs of our patients and healthcare providers continue to change, and we must change with them.”

As part of this new promise, Convatec commits to strengthening support for patients who are facing challenging issues associated with their health condition by expanding support programs such as me+, which provides educational and guided recovery tools, tips, and peer‐to‐peer‐support to those living with long‐term health conditions.

Convatec is also dedicated to working hand‐in‐glove with patients, caregivers, and health care professionals to help prevent stigma. Convatec recently launched its first “Healthy Bonds” campaign, which celebrates, supports, and empowers people in the ostomy community, who often face stigma, by sharing stories of how they are embracing life with an ostomy.

At the same time, Convatec is steadfast in its commitment to lifting the burden from health care providers by providing educational resources and added assistance through programs like Convatec Academy of Professional Education, which is dedicated to supporting the ongoing education and training of health care professionals. The program has already engaged with more than 300 000 health care professionals globally in the past year.

Convatec also realises that the time of nurses and health care professionals is valuable. To lift the burden off health care providers, Convatec launched a new nurse solutions app which makes it easier for them to select and recommend the best ostomy products for their patients, while also providing patients with a convenient one‐stop‐shop to fulfil their prescriptions, find advice and schedule a consultation with a nurse.

Since 1964, Convatec has long supported patients with needs for the management of long‐term health conditions, with leading market positions in advanced wound care, ostomy, and infusion care. “Forever caring” represents a new and exciting stage of Convatec's history, as well as a promise to help give patients and health care providers the support they need.

